# Primary care consultations on emotional distress – a part of the acculturation process in patients with refugee backgrounds: a grounded theory approach

**DOI:** 10.1186/s12875-021-01487-9

**Published:** 2021-06-30

**Authors:** Erica Rothlind, Uno Fors, Helena Salminen, Per Wändell, Solvig Ekblad

**Affiliations:** 1 Culture Medicine, Department of Learning, Informatics, Management and Ethics, Karolinska Institutet, Stockholm, Sweden; 2 Department of Computer and Systems Sciences, Stockholm University, Stockholm, Sweden; 3 Department of Neurobiology, Care Sciences and Society, Division of Family Medicine and Primary Care, Karolinska Institutet, Huddinge, Sweden; 4 Academic Primary Health Care Centre, Region, Stockholm, Sweden

**Keywords:** General practice, Primary care physicians, Qualitative research, Culture, Acculturation, Mental disorders

## Abstract

**Background:**

Considering the global refugee crisis, there is an increasing demand on primary care physicians to be able to adequately assess and address the health care needs of individual refugees, including both the somatic and psychiatric spectra. Meanwhile, intercultural consultations are often described as challenging, and studies exploring physician–patient communication focusing on emotional distress are lacking. Therefore, the aim was to explore physician–patient communication, with focus on cultural aspects of emotional distress in intercultural primary care consultations, using a grounded theory approach, considering both the physician’s and the patient’s perspective.

**Methods:**

The study was set in Region Stockholm, Sweden. In total, 23 individual interviews and 3 focus groups were conducted. Resident physicians in family medicine and patients with refugee backgrounds, originating from Somalia, Syria, Afghanistan and Iraq, were included. Data was analysed using a grounded theory approach.

**Results:**

Over time, primary care patients with refugee backgrounds seemed to adopt a culturally congruent model of emotional distress. Gradual acceptance of psychiatric diagnoses as explanatory models for distress and suffering was noted, which is in line with current tendencies in Sweden. This acculturation might be influenced by the physician. Three possible approaches used by residents in intercultural consultations were identified: “biomedical”, “didactic” and “compensatory”. They all indicated that diagnoses are culturally valid models to explain various forms of distress and may thus contribute to shifting patient perceptions of psychiatric diagnoses.

**Conclusions:**

Physicians working in Swedish primary care may influence patients’ acculturation process by inadvertently shifting their perceptions of psychiatric diagnoses. Residents expressed concerns, rather than confidence, in dealing with these issues. Focusing part of their training on how to address emotional distress in an intercultural context would likely be beneficial for all parties concerned.

**Supplementary Information:**

The online version contains supplementary material available at 10.1186/s12875-021-01487-9.

## Background

In the wake of the ongoing global refugee situation, where forcibly displaced people run greater risks of developing both somatic and psychiatric diseases, timely access to primary care services in the receiving countries is imperative [1, 2]. To facilitate access to treatment worldwide, mental health services are, in accordance with WHO recommendations, gradually being integrated into primary care [2]. For primary care physicians (PCPs) this implies an ability to deal with emotional distress, and adequately diagnose emotional disorders in an intercultural context [1].

The distinction between ‘emotional distress’ and ‘emotional disorder’ is unclear, since symptoms experienced and displayed overlap [3, 4]. There seems, however, to be an understanding that there is a difference in severity, disorder indicating a more severe condition, while distress is a milder, and not necessarily pathological, reaction [3, 5]. In this study, we use the term emotional distress to reflect that PCPs in general deal with a significant volume of sub-clinical presentations [5].

Refugee populations in general show a higher prevalence of various emotional disorders, such as depression [1, 2, 6–8]. This might be due partly to the process of migration itself, with exposure to pre-migration, migration and post-migration stressors [1, 2, 7–9]. However, concerns have also been raised regarding the risk of over-diagnosing and over-treating distress [5, 10]. It has also been argued that PCPs need to be more confident in dealing with sub-clinical mental health conditions, instead of using depression as an ‘all-inclusive’ diagnostic label [5].

Increasing the specificity of diagnosis is not least important in intercultural consultations as epidemiological research suggests that there is cultural variation in, for example, the symptomatology of depression [11, 12]. Taking the patient’s level of acculturation into account has also been suggested as valuable in diagnostic interviews [13, 14]. Acculturation is a concept originating from anthropology. It refers to changes taking place on behavioural and cognitive levels when people from different cultures meet [4]. A more comprehensive discussion of our understanding of the concept of culture has been published elsewhere [15].

To date, studies of intercultural consultation in primary care have focused largely on experiences of consultations in general. Various communicative difficulties were recurring themes [16–19]. In one study, a model called ‘circling the undefined’ illustrated *why* this might be the case; since differing explanatory models of disease are seldom acknowledged, the core of the problem is often left unaddressed [15]. Intercultural training may improve physicians’ knowledge, but there is a need to address behavioural outcomes as well [20]. A systematic review synthesising challenges and facilitators, as identified by health professionals in primary care, labelled one challenge ‘cultural understanding’[21]. This included ‘different understandings of health concepts and terminology’ [21].

Mental health literacy is a concept to consider in this context. It has been defined as ‘knowledge and beliefs about mental disorders which aid their recognition, management or prevention’ [22]. It has also been described as a ‘Western scientific conceptualization’ [23] that may challenge traditional beliefs, and efforts to improve mental health literacy need to consider the influence of culture to be successful [23]. Studies have focused on large-scale interventions, for example community campaigns, rather than what takes place on an interpersonal level in the consultation [23].

In summary, intercultural consultations in general are perceived as complex, not least due to communicative issues and different understandings of concepts related to health and disease. In consultations dealing with emotional distress and disorders, adequate communication is essential, as the foundation for the diagnostic process and appropriate care [24, 25]. Nonetheless, qualitative studies focusing specifically on physician–patient communication on emotional distress in intercultural primary care consultations are lacking. Culture is also a concept included in previously identified challenges [21]. However, the acculturation process has, as far as we know, not been explored in the context of intercultural primary care consultations. Even though part of the process might be taking place here, given repeated meetings.

Therefore, the aim of this study was to explore physician–patient communication, with focus on cultural aspects of emotional distress in intercultural primary care consultations. We used a grounded theory approach, considering both the physician’s and the patient’s perspective.

## Methods

The study used a grounded theory (GT) approach as outlined by Charmaz [26], who advocates a more ‘constructivist’ approach than the original developers [27]. This approach was chosen as the study is based on the theoretical framework of symbolic interactionism [28]. A key feature of symbolic interactionism is that symbols, such as language, play significant parts in shaping our understanding of reality [26, 28]. Within a group of people, the meaning, or understanding, of a specific symbol (or word) is shared. But when interacting with others, this understanding may be re-interpreted, and a new meaning is co-constructed. Since our understanding of culture as something dynamic, constructed in social interactions, is in line with symbolic interactionism, we found it suitable as a framework for addressing the aim of the study.

### Setting and context

The setting was Swedish primary care, with informants recruited from primary care centres (PCCs) in various socio-economic contexts in Region Stockholm.

In Sweden, primary care is in general the first point of contact for patients with emotional distress and disorders, this applies to the population in general, as well as to refugees [29]. Sweden is a recipient European country with a relatively high number of refugees per capita [30, 31]. It also has a high estimate of emotional disorder in the general population: thus in 2018 approximately ten percent had received a prescribed anti-depressant [32]. The Swedish health care system and its organisation of primary care are reviewed in detail in previously published material [29].

### Recruitment and description of informants

Informants were recruited using theoretical sampling [26]. The process allows for parallel recruitment/data collection and analysis to permit exploration of new themes as they emerge, aiming to achieve rich data and theoretical saturation; that is, until new information no longer contributes to new theoretical insights [26]. By using this process of recruitment, we were able to strive for diversity in terms of for example gender, nationalities and years living in Sweden.

The informants were 15 postgraduate physician trainees (residents) in family medicine and 23 patients with refugee backgrounds. The inclusion criterion for the residents was a current residency in family medicine; no exclusion criteria were applied. The residents were contacted by e-mail using a list provided by the regional centre for resident education in primary care. It consisted of 60 residents enrolled in a mandatory course in research methodology. In total, 10 men and 5 women were included. The median period as a resident was 5 years (range 3–9), 8 had a medical degree from Sweden and 7 had attended medical school abroad. We included residents, instead of specialists, believing that their accounts would enable us to identify issues calling for improved educational interventions.

The inclusion criteria for the patients were ages 18–65 years, a refugee background (as identified by themselves), and a Swedish residence permit. Asylum-seekers and undocumented migrants were excluded due to their insecure circumstances. The patients were invited to participate through written information distributed by staff at three PCCs. The 23 patients included originated from Somalia (n = 13), Syria (n = 5), Afghanistan (n = 4), and Iraq (n = 1). Median age was 40.5 years (range 23–65), and median years in Sweden was 8 (range 1–27). The level of education among the patients was evenly distributed between primary school (n = 7), secondary school (n = 8), and higher education (n = 8). All patients were offered an interpreter, but the majority (n = 17) declined. When interpreters were used, translation-checking was applied in order to verify accuracy [33]. Although the interpreters occasionally gave a briefer account, the contents did not change.

### Data collection and analysis

Data was collected between 2015 and 2017 through individual and focus group interviews (FGI). Locations for the interviews were chosen by the informants and included, for example, meeting facilities provided by Karolinska Institutet and local libraries. The residents were interviewed individually (n = 15), some of the patients individually (n = 8), and some in 3 focus groups with 4–7 informants, each of mixed gender. Although the FGIs were conducted in Swedish, the informants in the respective groups had the same native language. Mean duration of the interviews with the residents was 58 min, the individual patient interviews 51 min and the FGIs 71 min.

The reason for using both individual interviews and FGIs was to explore whether, as is sometimes the case, informant interaction would generate new information [34, 35]. In comparison with individual interviews, there are some additional difficulties that need to be considered in FGIs. Having skilled researchers conducting the FGIs is imperative. In this instance ER and SE, both experienced in conducting FGIs, were present. ER as moderator and SE with the role of note-taking observer, whose role it is to capture group processes. The interaction of the informants is a vital part of the data; hence it is important not to let a dominant individual set the tone for the whole group, impeding the participation of the others. This is a well-known pitfall which can be prevented using an experienced moderator and careful planning prior to the FGI; for example, power balance and the size of the group demand consideration [36]. Although challenges exist, FGIs have also been suggested as means to engage informants from culturally diverse backgrounds in research to a greater extent [36]. One reason being that informants may feel more comfortable discussing experiences with similar others, rather than with a researcher [35, 36].

In general, richer accounts were given in the FGIs, permitting theoretical saturation of previously emerged categories.

The individual interviews were conducted by master-level medical students, trained in interviewing skills, and supervised by authors ER and SE. The main reason for this delegation was that the authors’ positions (specialist in family medicine and psychologist with focus on intercultural health care) might have prevented the informants from raising criticism and/or uncertainties. As the researcher is part of the process in GT, we found this possible power imbalance important to take into consideration [26].

Interview guides were developed, and pilot tested; one for the residents and one for the patients. In accordance with GT, open questions were used. When planning interview guides, particular care must be taken when informants from culturally diverse backgrounds are involved, as nuances may be lost [36]. There is a delicate balance between using questions that are too vague to yield meaningful answer and using leading questions. Follow-up questions asking for specific examples are useful in this context and were used frequently. This is part of the semi-structured interview technique used which, in accordance with GT methodology, allows for the exploration of new areas of interest along the process [26]. Since discussions on emotional distress recurred early on, it was added as a separate topic. A guide of the topics covered is provided in Additional file 1.

Data was collected and analysed in parallel, and each interview was audiotaped, transcribed and analysed prior to the next [26]. Field notes and memos were written after the interviews and used as tools for analysis and reflection. The process of analysis used initial, focused coding and categorising in accordance with GT as outlined by Charmaz [26]. Initial codes were in general of a descriptive nature, sticking close to the data. Focused coding involved assessing the initial codes, through comparison with each other and against an increasing amount of data and moving forward with those deemed to have more theoretical reach. These were sorted into more substantive, although preliminary, categories. The iterative process of data collection and analysis was carried out until theoretical saturation was reached [26]. One example to illustrate the coding process is the following quote:

Then I try to explain that it’s just like any other substance in your body, if you get too little insulin, you get a disease called diabetes, if you get too little serotonin you get a depression [...] *(authors’ translation)*

The analysis generated the following coding steps: ‘explaining depression through comparison with diabetes’ – ‘somatic similes used to inform about psychiatric diagnoses’ – ‘the didactic approach’. The didactic approach was one of the sub-categories. It involved the residents teaching the patients about various diagnoses (explained further under Results). The coding was primarily carried out by ER and SE, first independently, then in discussions with the other co-authors. Although the coding was done by hand, the codes were organised and stored using the software NVivo.

To reduce the risk of misinterpretation, triangulation was applied using multiple researchers, a combination of individual interviews and FGIs and by including both a physician and a patient perspective. Relevance of the results was confirmed through what is called member-checking [26], where both physicians and patients with refugee backgrounds were presented with preliminary results in focus-group settings. No categories were changed.

### Ethics

The study was approved by the Regional Ethical Review Board in Stockholm (2015/1228–31/5 and 2016–2308-32). For the informants, the main risk identified was sharing sensitive information. To ensure that informants were not identifiable, quotes were selected with care and transcripts were anonymised. Extra consideration was given to the patients included, as on a group-level, foreign-born patients are often considered at risk of disadvantage. On the other hand, care should be taken to include groups underrepresented in research, in accordance with the ethical principles of medical research, as outlined in the Declaration of Helsinki [37]. In addition, their specific experience was needed to address the identified gap of knowledge. They were also informed that if any concerns were raised, they could contact either ER or SE, and if needed appropriate referral would be arranged (both are working clinically with similar issues).

All informants received written and verbal information on the purpose of the study. The written information for the patients was translated and back-translated [38] by professional translators. Confidentiality and the voluntary nature of participation, with the right to decline or withdraw at any time without giving reasons, were emphasised. Informed consent was given in writing by all informants.

## Results

### Primary care consultations on emotional distress as part of acculturation – a conceptual model

**Fig. 1:**
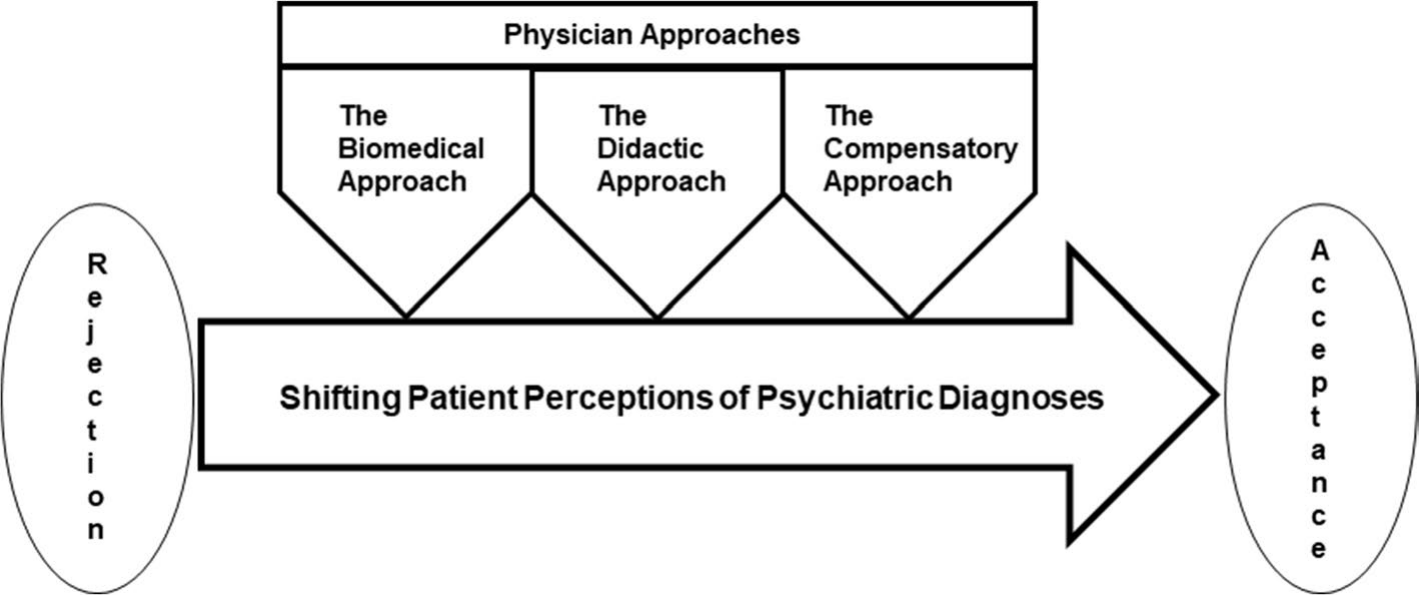
A conceptual model describing how primary care consultations on emotional distress constitute part of the acculturation process. This schematic figure, derived through a grounded theory approach, illustrates that a shift in patient perceptions of psychiatric diagnoses might be influenced by various physician approaches (one or all described), with no chronological order inferred

The resident physicians interviewed described difficulties in getting newly arrived patients with foreign background to accept psychiatric diagnoses as explanatory models of illness. However, when seeing the patient over time, they often noticed a gradual shift towards acceptance. This was also reflected in the patients’ accounts, and in their reactions to questions on the topic during the interviews. Our conceptual model ‘Primary care consultations on emotional distress as part of acculturation’, (Fig. 1), illustrates how three physician-approaches (sub-categories) contribute to our main category of ‘Shifting patient perceptions of psychiatric diagnoses’. The sub-categories were labelled 1) the biomedical approach, 2) the didactic approach, and 3) the compensatory approach.

The first sub-category describes residents trying to solve non-medical issues using a biomedical approach. The second sub-category incorporates active teaching of explanatory models of illness. The third sub-category outlines how residents used a compensatory approach in trying to alleviate suffering. The main category and three sub-categories are described below, including examples of de-identified quotations.

### Main category: Shifting patient perceptions of psychiatric diagnoses

Shifting patient perceptions of psychiatric diagnoses was identified as our main category. With time, patients seemed to move gradually from rejection to acceptance of psychiatric diagnoses as valid models for explaining illness, as described from both the resident’s and the patient’s perspective in our interviews.

The residents described how they felt they were often met with resistance from newly arrived patients with foreign backgrounds, when they suggested psychiatric diagnoses and related treatments. ‘Stigma’ and’taboo’ were terms used as possible explanations.

Another problem is to refer them [refugees] to a psychologist or psychiatrist since they are stigmatising, a cultural stigma you might say. (Res14) *(authors’ translation)*

With time, however, they described how a change in the patient could occur.

Patients come with vague problems and gradually after a certain number of visits these have sort of got converged into a symptomatology that is some kind of compromise between the patient’s symptoms and our diagnostics. (Res8) *(authors’ translation)*

The patients’ accounts also reflected this shift. Patients who had lived in Sweden for a while described how, initially, the meaning of terms such as depression and anxiety was not clear to them, but with time they felt they had gained knowledge.

I think those of us sitting here today we understand, sometimes you can have a light depression and then you will be ok […] We understand this and we can discuss it, but the newly arrived don’t. (Pat2FGI3) *(authors’ translation)*

The patients described how initially they perceived it as strange and intrusive to be asked by PCPs about symptoms of distress. However, they had come to learn that emotional distress was not seen as a stigma, rather it was something commonly discussed in primary care consultations.

Where I come from, talking about emotional distress is taboo […] and the physician would never ask about it […] But now when we have lived here [in Sweden] we have learned that here physicians ask different questions (Pat6) *(authors’ translation)*

This gradual shift was also reflected in the patients’ behaviour during our interviews. When asked, in a neutral way, about their views in general on, for example, primary care being responsible for treatment of emotional distress, the response varied greatly. At one end of the spectrum, it was seen as an almost offensive question and the responses clearly stated it was an off-limits topic. At the other end, however, emotional distress was expanded upon and discussed in a reflective manner, at great self-distance, also with humour.

So, I asked my mother the other day: ‘How come jinns *(evil spirits, authors’ comment)* only affect Somalis?’ She’s like: ‘Oh be quiet’ (Pat3FGI3) *(authors’ translation)*

In summary, a shift in patient perceptions from rejection towards acceptance of psychiatric diagnoses as culturally valid models to explain suffering and illness emerged from the analysis.

Analysing the residents’ possible role in this process, we identified three possible approaches. Outlined below, these approaches constitute our sub-categories. Some residents also expressed doubts in the interviews regarding these approaches, their concerns are also incorporated under each heading.

#### Sub-category 1: The biomedical approach

The residents described dichotomising patients’ problems into either a medical or a non-medical category. A view shared by some of the patients.

When it comes to emotional distress and things like that, I don’t think there is anything [the physician] can do about it, because it’s caused by war and that is something you can’t do anything about. (Pat7) *(authors’ translation)*

In consultations with patients with refugee backgrounds, the residents described non-medical problems as often being dominant. Dealing with non-medical issues was not something they had been trained to do, nor in general felt was a part of their job.

But people often come to the doctor and then it’s really a social problem and it’s terribly hard to deal with since we really have so little to do with it. (Res3) *(authors’ translation)*

This could, however, result in a biomedical approach being applied when trying to solve non-medical problems, which almost by default would involve a diagnosis.

If they [the patients] meet diagnostic criteria, it’s easier to send them on to psychiatry. […] The important thing is to meet [diagnostic] criteria. (Res12) *(authors’ translation)*

Consequently, if seeking medical advice alone, the patient may risk being diagnostically labelled. Indirectly, the message communicated to the patients is that diagnoses are culturally valid models to explain suffering.

***Concerns expressed:*** This dualistic approach was at the same time questioned by some of the residents, as it was clear to them that the patients’ strained social circumstances often affected their well-being. But even so, it was not evident how this knowledge could be applied in the consultation, or if it could be incorporated into a biomedical assessment.

If someone told me “you can’t work as a doctor now, you have to be a cleaner” I’d feel really bad and this is [an example of] when you feel that “this is, kind of, not medical … or is it? Do they have a psychiatric diagnosis?” (Res1) *(authors’ translation)*

#### Sub-category 2: The didactic approach

The residents noted that using psychiatric diagnoses as explanatory models of illness was at first often met with reluctance and/or puzzlement from patients. Some interpreted this as a result of poor mental health literacy and responded accordingly with a didactic approach (or a ‘crash course’ as mentioned by one resident). Methods used varied but making comparisons with somatic diseases was a recurring example.

Some of them don’t understand: ”What does depressed mean?” Then I try to explain that it’s just like any other substance in your body: if you get too little insulin, you get a disease called diabetes, if you get too little serotonin, you get a depression. (Res4) *(authors’ translation)*

A common goal of the didactic approach seemed to be ‘normalisation’ of psychiatric diagnoses, and again, the message communicated to the patient is that diagnoses are culturally valid models to explain suffering. The process of normalisation was also reflected in the patient interviews, the following quote is one example.

Then [when I was newly arrived], I didn’t understand these things […] but here [in Sweden] they have knowledge about things like depression, I mean they accept politicians taking timeout to recover from burnout. (Pat3FGI1) *(authors’ translation)*

***Concerns expressed:*** Some of the residents did however show awareness of cultural differences in how emotional distress could be expressed and questioned whether asserting ‘their’ nomenclature would be useful to the patient. Questions were also raised as to whether some diagnoses were even applicable worldwide, and if so, would they mean the same thing to the individual?

I think it’s terribly difficult; if I say anxiety, […] is there even a word for anxiety? What does the patient think when I say anxiety? (Res2) *(authors’ translation)*

#### Sub-category 3: The compensatory approach

The compensatory approach involves medical assessment of the patient as influenced by misguided sympathy to the extent where objectivity might be compromised. The residents often found themselves responding to the patient’s exposed situation with an instant desire to help. Instead of maintaining a professional empathic approach, some would try to alleviate the patient’s suffering by means of compensation.

As a professional you cannot affect it [the patient’s exposed situation], and then again, I know many I’ve worked with who choose to write certificates […] on this or that which perhaps are not medical. (Res5) (*authors’ translation)*

In trying to compensate or help the patient, a limited number of tools are easily available to the physician, such as prescribing medication or issuing certificates for sick-leave or other benefits. Both require the patient to have a diagnosis. Thus, the patient is again likely to be diagnostically labelled, but whether the diagnosis is based on an objective medical assessment could be questioned.

***Concerns expressed:*** Questions on the theme of whether it is possible to medicate suffering were raised. Residents admitted prescribing both anti-depressants and sleep medication with the intention to help, while at the same time strongly doubting whether the patient would benefit.

I can’t solve the problem of your child living in another country. Is this going to help? No, perhaps you’ll sleep a bit better, but you won’t feel better mentally. (Res10) *(authors’ translation)*

A patient addressing the same issue said:

I don’t believe in things like talking to a psychologist, but still I need help. But the physician is going to say: “I’m just a physician, I can’t stay in touch with you all the time and I can’t comfort you either”. So, what can you do for me then? (Pat10FGI2) *(authors’ translation)*

## Discussion

### Statement of principal findings

The study aimed to explore physician–patient communication, with focus on cultural aspects of emotional distress in intercultural primary care consultations. Our findings show that resident physicians, in intercultural consultations, may be part of patients’ acculturation process in terms of influencing their perception of emotional distress, shifting from rejection to acceptance of psychiatric diagnoses as explanatory models.

Through the approaches described: the biomedical, the didactic, and the compensatory, the residents signalled that psychiatric diagnoses, in this cultural context, are valid explanatory models of distress. Patients could thereby be guided to a restructured framework of understanding suffering and distress. Our main category was therefore labelled ‘Shifting patient perceptions of psychiatric diagnoses’. While this could be interpreted as a result of improved mental health literacy, we do believe this interpretation would neglect the influence of cultural aspects. During the interviews, the residents raised questions as to whether this shift was beneficial to the patients, suggesting a complexity beyond increasing mental health literacy levels. These concerns did not, however, seem to be voiced to any extent in the clinic. A lack of opportunity to discuss and openly reflect on these concerns may contribute to diagnostic labelling being the main path of action.

### Findings in relation to previously published work

The understanding of emotional distress and its implications varies across cultures; this has been established [10]. This study contributes with novel findings in suggesting how a process of changing this understanding takes place in patients, over time, when they are exposed to another culture, through interactions with PCPs.

The findings will be discussed largely against the backdrop of our theoretical approach of symbolic interactionism, which emphasises social interactions in forming one’s understanding of reality [26]. This approach is also reflected in the concepts of ‘emotional acculturation’[39], and ‘explanatory models’ [40], which are discussed below in relation to our findings.

Emotional acculturation is a concept we believe provide a possible framework for comprehending the shift from rejection to acceptance described in our model. Research in psychology and anthropology suggests that emotional experience is culturally constructed [41, 42]. Across cultures, emotions that are considered normative differ; when comparing patterns of emotions, individuals generally make a better fit with the average pattern of their own cultural group than that of others [41, 42]. For migrants this could mean that the emotional patterns considered normative in their culture of origin, might not be the same as those valued in their new cultural context. Thus, they might risk facing a ‘cultural mismatch’ [42]. However, when individuals are exposed to another culture, over time, patterns of emotions can change – this process of change was relatively recently (2011) coined ‘emotional acculturation’ [39]. We suggest that emotional acculturation partly explains why patients, as described by one of the residents interviewed, ‘converge into a symptomatology’. The degree to which this process occurs seems to be predicted by the amount of exposure to the majority culture; both via the proportion of life spent in the new culture, and via interpersonal interactions with majority members of that culture [39, 42].

Considering the idea that interpersonal interactions drive the process of emotional acculturation, where each meeting may produce successive changes in emotional patterns [41], we suggest that patient-physician consultation is not an exception. As illustrated by our results, the consultation does not take place in a cultural vacuum, residents are influenced both by their own macro-culture and by their micro-culture. In this context, it could be argued that both micro-, and macro-cultures may facilitate diagnostic labelling of suffering. The residents’ micro-culture is likely to be dominated by their biomedical training, which advocates the use of diagnosis in general. The macro-culture in Sweden, as in several other parts of ‘the Western world’, has gradually shifted from stigmatising emotional distress, to embracing diagnoses as explanatory models of suffering and distress, while in other parts of the world this is not the case [10]. According to the results, a mismatch in explanatory models could be perceived by the residents, as a lack of mental health literacy and adjusted for through the didactic approach. This is in line with the concept of explanatory models as outlined by Kleinman, where eliciting the patient’s model and, if necessary, aligning it with the physician’s, is central; teaching being part of the process [40]. Just as interventions aiming to improve mental health literacy are recommended to consider cultural aspects [23], ‘teaching’ the patient as part of the consultation also needs to be done in a culturally sensitive manner not to risk alienation.

The same issue has been addressed on a global scale. It has, for example, been questioned whether standard modes of diagnosis and interventions existing in one part of the world are applicable in different cultural contexts [43]. An example of a culturally adapted intervention, used in Zimbabwe, is the Friendship Bench [44]. It is an intervention applying problem-solving therapy, which instead of a diagnosis focuses on a problem, identified by the patient. Evaluating the intervention, the significance of having health care workers who were perceived as culturally relevant by the community, was emphasised [44].

Challenges of the diagnostic procedure in an intercultural context, have also been highlighted in previous research in psychiatric settings. One example is how to separate culturally ‘normal’ responses from responses indicating pathology [45]. A Swedish study interviewing patients with immigrant backgrounds, who had received treatment in various psychiatric settings, introduced the concept of ‘restructuring illness meaning’, which included ‘a push toward giving illness and suffering a psychological or psychiatric meaning’ [46]. The patients described the process of restructuring as having been a ‘disruptive experience’ [46].

That it could be problematic to assert psychiatric nomenclature, mirrors the concerns voiced by the residents in our study. In summary, these are reasonable concerns, when combining the process of emotional acculturation with the described approaches facilitating diagnostic labelling, there is likely a non-negligible risk of contributing to the creation of patients, instead of strengthening a person’s coping mechanisms.

### Strengths and weaknesses

In a grounded theory study, the interaction between the researchers, the informants and the data will always affect the result; this is one of the main issues needing to be dealt with [26]. One approach, applied in this study, is to continuously reflect on how one’s own beliefs and preconceptions influence the data, through for example group sessions and the use of memos.

The main strength of the study was the diversity of the informants (both inter- and intra-group), which resulted in saturated data, illuminating the same issues but from different perspectives. A dual perspective enriching the data was also added by some of the residents having foreign backgrounds. Letting the patients define themselves as having a refugee background also added to the heterogeneity of the group. However, we do realise it might be considered a weakness that we did not confirm their status. On the other hand, doing so would not be in line with our ethical standpoint.

Language barriers were considered the main limitation. Although most informants were proficient in Swedish, some interviews required interpreters. Not only is the researcher then dependent on the skills of the interpreter, inevitably the presence of a third-party will also change the preconditions for the interview [47, 48]. Issues such as trust and confidentiality may be compromised, and the information disclosed may be altered [47, 48]. By using professional interpreters and applying translation-checking we tried to reduce the risk of misconstructions. Also, when analysing the interviews, we noted no recurring differences; negative and positive statements were expressed by both groups.

We comment briefly on the use of the concept of ‘time’ in the manuscript, as this could be considered a weakness. While we recognise that temporal phrases such as ‘over time’ are imprecise, the format and focus of this qualitative study did not allow for any more precise wordings. The time required for the shifting views described is also likely to be highly individual, and therefore maybe not even possible to estimate.

The criteria of fit, relevance, workability and modifiability are often used when evaluating GT studies [26, 27]. Fit means that empirical data is directly reflected in the results [26, 27]. Collecting and analysing data in parallel, as in this study, is one way of ensuring this. Relevance of the results was confirmed by member-checking. Making observations in a clinical setting would be of value to check the workability and modifiability of the results. It is intended to do this in future studies.

### Implications

Our results indicate that resident physicians share in patients’ acculturation processes when it comes to changing the latter’s perception of emotional distress. If more studies confirm the results, we believe that raising awareness of the PCP’s possible role in patient acculturation, and how to address this, should be considered part of residents’ training in intercultural consultation. The importance of contributing to increased mental health literacy should not be overlooked, but there is a need to address how to achieve this in intercultural consultations dealing with emotional distress.

## Conclusion

Being a part of intercultural consultations as a primary care physician (PCP), also means taking part in the patient’s acculturation process in terms of changing their perceptions of emotional distress. Estimating how far this takes place in the individual case is not possible. Viewing it as something positive or negative is probably too simplistic. However, we do believe it is significant for PCPs to realise that they are likely to play a substantial part. While resident physicians might be trained in learning criteria for depression, they might not be given time to reflect upon how their own cultural patterns affect the outcome of the consultation. They are trained to deal with well-defined diseases from a biomedical approach, but they need to be better prepared to address suffering and distress in an intercultural context.

## Supplementary Information

**Additional file 1.** Topic guide for the interviews.

## Data Availability

Transcripts of the interviews analysed are not publicly available due to the risk of compromising individual privacy, but data is available from the corresponding author on reasonable request. The topic guide used is submitted as an additional file.

## References

[1] Silove D, Ventevogel P, Rees S (2017). The contemporary refugee crisis: an overview of mental health challenges. World Psych.

[2] Priebe S, Giacco D, El-Nagib R (2016). WHO health evidence network synthesis reports. Public health aspects of mental health among migrants and refugees: a review of the evidence on mental health care for refugees, Asylum seekers and irregular migrants in the WHO European Region.

[3] Hassan G, Kirmayer LJ, Mekki-Berrada A, Quosh C, el Chammay R, Deville-Stoetzel J (2015). Culture, context and the mental health and psychosocial wellbeing of Syrians: a review for mental health and psychosocial support staff working with Syrians affected by armed conflict.

[4] Berry JW (1997). Immigration, acculturation, and adaptation. Appl Psychol.

[5] Goldberg SD (2019). Are official psychiatric classification systems for mental disorders suitable for use in primary care?. Brit J Gen Pract J Royal College Gen Practitioners.

[6] Fazel M, Wheeler J, Danesh J (2005). Prevalence of serious mental disorder in 7000 refugees resettled in western countries: a systematic review. Lancet.

[7] Giacco D, Laxhman N, Priebe S (2018). Prevalence of and risk factors for mental disorders in refugees. Semin Cell Dev Biol.

[8] Bogic M, Njoku A, Priebe S (2015). Long-term mental health of war-refugees: a systematic literature review. BMC Int Health Hum Rights.

[9] Lindencrona F, Ekblad S, Hauff E (2008). Mental health of recently resettled refugees from the Middle East in Sweden: the impact of pre-resettlement trauma, resettlement stress and capacity to handle stress. Soc Psychiatry Psychiatr Epidemiol.

[10] Kirmayer LJ, Gomez-Carrillo A, Veissiere S (1982). Culture and depression in global mental health: An ecosocial approach to the phenomenology of psychiatric disorders. Soc Sci Med.

[11] Haroz EE, Ritchey M, Bass JK, Kohrt BA, Augustinavicius J, Michalopoulos L (2017). How is depression experienced around the world? A systematic review of qualitative literature. Soc Sci Med.

[12] Kessler RC, Bromet EJ (2013). The epidemiology of depression across cultures. Annu Rev Public Health.

[13] Alarcon RD (2009). Culture, cultural factors and psychiatric diagnosis: review and projections. World Psych.

[14] Yoon E, Chang CT, Kim S, Clawson A, Cleary SE, Hansen M (2013). A meta-analysis of acculturation/enculturation and mental health. J Couns Psychol.

[15] Rothlind E, Fors U, Salminen H, Wandell P, Ekblad S (2018). Circling the undefined-A grounded theory study of intercultural consultations in Swedish primary care. PLoS ONE.

[16] Jensen NK, Norredam M, Priebe S, Krasnik A (2013). How do general practitioners experience providing care to refugees with mental health problems? A qualitative study from Denmark. BMC Fam Pract.

[17] Wachtler C, Brorsson A, Troein M (2006). Meeting and treating cultural difference in primary care: a qualitative interview study. Fam Pract.

[18] Papic O, Malak Z, Rosenberg E (2012). Survey of family physicians&apos; perspectives on management of immigrant patients: attitudes, barriers, strategies, and training needs. Patient Educ Couns.

[19] Baarnhielm S, Ekblad S (2008). Introducing a psychological agenda for understanding somatic symptoms–an area of conflict for clinicians in relation to patients in a multicultural community. Cult Med Psychiatry.

[20] Jongen C, McCalman J, Bainbridge R (2018). Health workforce cultural competency interventions: a systematic scoping review. BMC Health Serv Res.

[21] Robertshaw L, Dhesi S, Jones LL (2017). Challenges and facilitators for health professionals providing primary healthcare for refugees and asylum seekers in high-income countries: a systematic review and thematic synthesis of qualitative research. BMJ Open.

[22] Jorm AF, Korten AE, Jacomb PA, Christensen H, Rodgers B, Pollitt P (1997). &quot;Mental health literacy&quot;: a survey of the public&apos;s ability to recognise mental disorders and their beliefs about the effectiveness of treatment. Med J Aust.

[23] Jorm AF (2012). Mental health literacy: empowering the community to take action for better mental health. Am Psychol.

[24] Priebe S, Matanov A, Barros H, Canavan R, Gabor E, Greacen T (2013). Mental health-care provision for marginalized groups across Europe: findings from the PROMO study. Eur J Pub Health.

[25] Priebe S, Sandhu S, Dias S, Gaddini A, Greacen T, Ioannidis E (2011). Good practice in health care for migrants: views and experiences of care professionals in 16 European countries. BMC Public Health.

[26] Charmaz K (2014). Constructing grounded theory.

[27] Glaser BG, Strauss AL (1967). The discovery of grounded theory: strategies for qualitative research.

[28] Mead GH (1934). Mind, self and society.

[29] Anell A, Glenngard AH, Merkur S (2012). Sweden: health system review. Health Syst Transit.

[30] Summary of Population Statistics 1960–2019 [Internet]. Statistics Sweden. 2020 [cited 2020–09–22]. Available from: https://www.scb.se/en/finding-statistics/statistics-by-subject-area/population/population-composition/population-statistics/pong/tables-and-graphs/yearly-statistics--the-whole-country/summary-of-population-statistics/.

[31] The European Migration Network. EMN Annual Report on Migration and Asylum 2017–Sweden. Stockholm: Swedish Migration Agency; 2018.

[32] The National Board of Health and Welfare. Statistics on pharmaceuticals 2018. Stockholm: The National Board of Health and Welfare; 2019.

[33] Ko L (2011). Translation checking: a view from the translation market. Perspectives.

[34] Barbour RS, Kitzinger J (1999). Developing focus group research : politics, theory and practice.

[35] Ekblad S, Bäärnhielm S (2002). Focus group interview research in transcultural psychiatry: Reflections on research experiences. Transcult Psychiatry.

[36] Halcomb EJ, Gholizadeh L, DiGiacomo M, Phillips J, Davidson PM (2007). Literature review: considerations in undertaking focus group research with culturally and linguistically diverse groups. J Clin Nurs.

[37] World Medical Association Declaration of Helsinki (2001). Ethical principles for medical research involving human subjects. Bull World Health Organ.

[38] Chen HY, Boore JR (2010). Translation and back-translation in qualitative nursing research: methodological review. J Clin Nurs.

[39] De Leersnyder J, Mesquita B, Kim HS (2011). Where do my emotions belong? A study of immigrants&apos; emotional acculturation. Pers Soc Psychol Bull.

[40] Kleinman A, Eisenberg L, Good B (1978). Culture, illness, and care: clinical lessons from anthropologic and cross-cultural research. Ann Intern Med.

[41] Mesquita B, Boiger M, De Leersnyder J (2016). The cultural construction of emotions. Curr Opin Psychol.

[42] De Leersnyder J (2017). Emotional acculturation: a first review. Curr Opin Psychol.

[43] Rose N (2019). Our psychiatric future: the politics of mental health.

[44] Chibanda D, Weiss HA, Verhey R, Simms V, Munjoma R, Rusakaniko S (2016). Effect of a primary care-based psychological intervention on symptoms of common mental disorders in Zimbabwe: A randomized clinical trial. JAMA.

[45] Sandhu S, Bjerre NV, Dauvrin M, Dias S, Gaddini A, Greacen T (2013). Experiences with treating immigrants: a qualitative study in mental health services across 16 European countries. Soc Psychiatry Psychiatr Epidemiol.

[46] Baarnhielm S (2004). Restructuring illness meaning through the clinical encounter: a process of disruption and coherence. Cult Med Psychiatry.

[47] Bjork Bramberg E, Dahlberg K (2013). Interpreters in cross-cultural interviews: a three-way coconstruction of data. Qual Health Res.

[48] Ingvarsdotter K, Johnsdotter S, Ostman M (2012). Lost in interpretation: the use of interpreters in research on mental ill health. Int J Soc Psychiatry.

