# Supplementary material for: Primary care consultations on emotional distress – a part of the acculturation process in patients with refugee backgrounds: a grounded theory approach
Source: BMC Fam Pract. 2021 Jun 30;22:138. doi: 10.1186/s12875-021-01487-9 (PMC8247242; doi:10.1186/s12875-021-01487-9)
Supplement: Supplementary file 1 — Additional file 1. Topic guide for the interviews. [file 12875_2021_1487_MOESM1_ESM.docx]

# Topic guide

Focus on primary care physician-patient communication

## Patient interviews

INTRO: What is the first thing that comes to mind when you think about consulting a primary care physician?

Experiences of consultations with primary care physicians in Sweden

Differences/similarities between consultations in country of origin (other countries of residence) and Sweden

Turning to a physician vs turning to others (family, elder, religious leader/scripts etc) when and for what type of problems (Follow-up: when feeling distress, but not physically ill)

General views on psychiatric diagnoses (Follow-up: have they changed? If so, ask for examples)

Advice to newly arrived immigrant in Sweden/to physicians working with newly arrived immigrants

FINAL: additional comments and how the interview was perceived

## Resident interviews

INTRO: What is the first thing that comes to mind when thinking about intercultural consultations?

Experiences of consultations with patients with migrant backgrounds

Differences/similarities when comparing inter- vs intracultural consultations

Experience of treating patients (having migrant backgrounds) with emotional distress (Follow-up: how approached/discussed in the consultation)

General views on addressing/suggesting psychiatric diagnoses in intercultural consultations

Advice to newly examined colleague meeting/treating migrant patients with emotional distress

FINAL: additional comments and how the interview was perceived
